# Blood Vessel Density in Basal Cell Carcinomas and Benign Trichogenic Tumors as a Marker for Differential Diagnosis in Dermatopathology

**DOI:** 10.1155/2011/241382

**Published:** 2010-10-07

**Authors:** Julia Winter, Hermann Kneitz, Eva-B. Bröcker

**Affiliations:** Department of Dermatology, University Hospital Würzburg, Josef-Schneider-Strarße 2, 97080 Würzburg, Germany

## Abstract

In order to get insight into the density of blood vessels in the stroma of benign and malignant trichogenic neoplasms, immunohistological quantification of CD 31 positive vessels was performed in 112 tumors, comprised of 50 BCCs of nodular (35) or morphoeic (15) growth patterns, 17 Pinkus' tumors, as well as 17 trichoepitheliomas of which 6 were desmoplastic, 8 trichofolliculomas, and 20 trichoblastomas. *Methods*. Vessel density was counted within the tumors, in the tumor-surrounding stroma, and, as a control, in the normal skin of the operation specimen. The results were compared using statistical methods. *Results*. Whereas, irrespective of the patients' age and location of tumors, the vessel density in normal skin showed no significant differences (8.8 ± 2.7), the counts in the peritumoral stroma revealed significant differences between the different tumors investigated. The highest counts were obtained in BCC (24.7 ± 6.7) and the lowest in benign trichogenic neoplasms (around 14) Pinkus' tumors revealed intermediate counts (19.7 ± 6.6). The vessel densities within the tumors were generally low, and no correlation to the dignity was found. *Conclusion*. Determination of blood vessel density in the peritumoral stroma may be an additional parameter for differential diagnosis of trichogenic tumors of uncertain dignity.

## 1. Introduction

The skin receives its blood supply via a deep dermal plexus and a superficial, subpapillar plexus. Growth of dermal vasculature mainly occurs during embryogenesis and is regulated—as detected since the 1980s—by a variety of soluble factors of angiogenesis and antiangiogenesis [1]. VEGF is known as the main proangiogenic factor, and important antiangiogenic factors are thrombospondin 1 and 2. The vessel shape and number stay constant as long as a balance of pro and antiangiogenic stimuli exists [2]. In 1971, Folkman proposed that tumor growth is dependent on angiogenesis [3]. Angiogenesis is also important in a variety of other diseases, with hypoxia and inflammation as known stimuli [4, 5]. Without angiogenesis, a tumor does not reach a size larger than 1-2 mm and also will not metastasise [6]. The so-called angiogenic switch is characterized by predomination of proangiogenic factors, followed by formation of new vessels [7, 8]. Higher levels of microvessel densities, as measured in histologic specimen, were found associated with adverse prognosis in a variety of tumor entities [9–13]. 

The aim of this study was the question as to whether microvessel density may be associated with the biological behaviour of trichogenic tumors arising in human skin, and, therefore, their immunohistological determination might facilitate differential diagnosis between BCCs and their histological simulators.

## 2. Material and Methods

The 112 tumors derived from the dermatopathologic archives of the institution. Tumors had been excised from 2000 to 2008. Data on diagnosis, on age and sex of patients and location of the lesions as well as normal skin of the excision specimen were available (Tables 1 and 2). The tumors chosen for study were 50 basal cell carcinomas (BCCs), of which were 35 nodular BCCs and 15 morphoeic BCCs; 17  Pinkus' tumors, 17  trichoepitheliomas of which 6 were of desmoplastic growth pattern, 8 trichofolliculomas, and 20 trichoblastomas (Table 2).

The specimens were formalin-fixed and paraffin embedded. Serial sections were stained with H&E and CD31 (Clone JC70A, Dako Denmark, dilution 1 : 30) following routine immunohistological methods using Aethylcarbazole as chromogen and hematoxyline as counterstain. Counting of CD31 positive vessel structures was performed with a 200-time magnification using a squaric grid. Counting in at least six and at most 10 fields was done separately in the normal skin of the excision tips, in the peritumoral stroma, and within the tumors. Statistical evaluation was performed using the program statistical package for social sciences, version 15.0.1 (SPSS). Boxplots were performed for the graphics. The values for peritumoral vessel densities underwent a single factorial variance analysis followed by a post-hoc test (Tukey-HSD). *P* ≤ .05 was chosen as level of significance. Clinical data were evaluated using Microsoft Office Excel 2003.

## 3. Results

### 3.1. Low Variation of Vessel Density in Normal Skin

**Figure 1:**
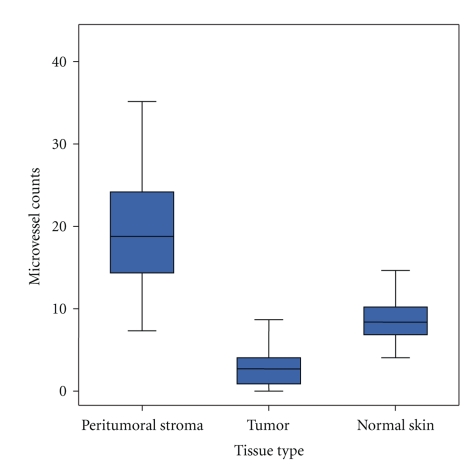
Boxplot for the microvessel counts in peritumoral stroma, tumor, and normal skin.

**Figure 2:**
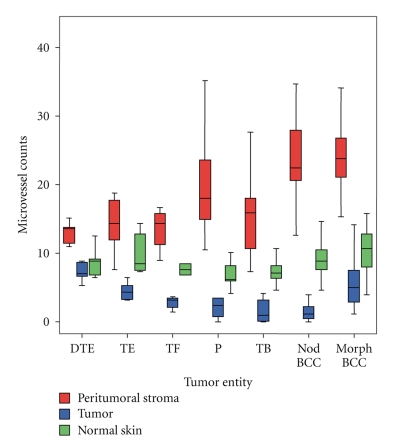
Boxplot for the microvessel counts in the tumor entities and tissue types.

Irrespective of the diagnosis, the location of lesions, and age/sex of patients, the vessel densities in the uninvolved skin of the operation specimen were rather low (8.8 ± 2.7) and did not vary significantly (Figures 1 and 2).

### 3.2. Low Intratumoral Vessel Density

The vessel density within the tumor tissue was surprisingly low—even lower than in normal skin—with mean values of 3.1 ± 2.9, irrespective of tumors dignity (Figures 1 and 2).

### 3.3. High Vessel Density in the Peritumoral Stroma of Malignant Tumors

**Figure 3:**
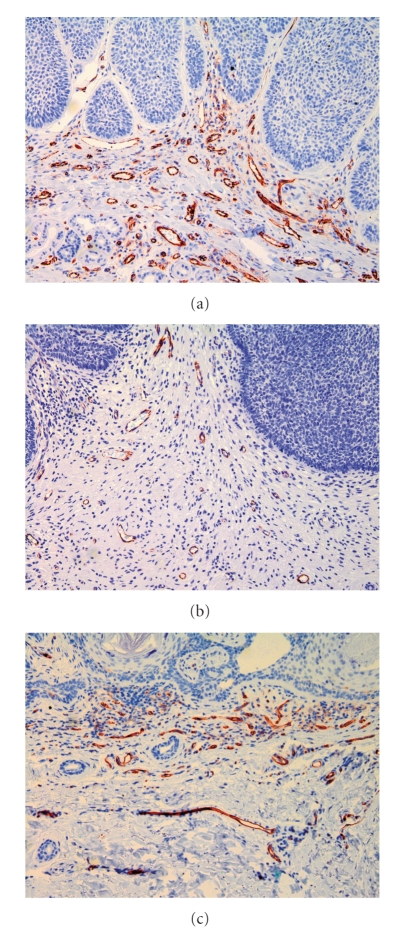
(a) CD 31 stained section of a nodular BCCs shows many vessels (200x magnification). (b) In a CD 31-stained section of a trichoblastoma, only a few vessels can be seen (200x magnification). (c) Microvessel density in a CD 31 stained section of a Pinkus' tumor is on an intermediate level (200x magnification).

In contrast to the generally low intratumoral vessel densities the number of CD 31 positive vessels in the tumor-surrounding stroma was higher (Figure 1), and showed a significant correlation to the tumors' dignity (Figure 2): Whereas nodular BCCs revealed 24.7 ± 6.7 peritumoral vessels/field the count for trichoblastomas—their benign counterparts—was 15.3 ± 5.1. This difference was highly significant (*P* < .0001). A CD 31-stained section of a nodular BCCs shows many vessels (Figure 3(a)) and CD 31 stained section of a trichoblastoma only a few vessels (Figure 3(b)). The Pinkus' tumors revealed intermediate counts (19.7 ± 6.6). Microvessel density in a CD 31 stained section of a Pinkus' tumor is on an intermediate level (Figure 3(c)). The mean counts and variations obtained in the different tumor entities are listed in Figure 2. Statistically significant differences were neither obtained between the different BCCs (nodular versus morphoeic) nor between all benign trichogenic tumors investigated.

## 4. Discussion

Angiogenesis is essential for tumor growth. This was already postulated by Rudolf Virchow in 1863 [14], and by Folkman in 1971 [3]. In a variety of human cancers, blood vessel density was found correlated to tumor aggressiveness and prognosis [15–18]. Angiogenesis and tumor vasculature serve as targets for innovative oncologic therapy regimen [19]. The aim of the study presented here was to evaluate whether blood vessel density as the result of former angiogenesis might reflect the biologic behaviour (dignity) of BCCs and benign cutaneous tumors derived from hair follicles. For this purpose, a rather large series of 112 benign and malignant trichogenic skin neoplasms was studied with regard to small blood vessel density in the tumor stroma. Noninvolved skin of the operation specimen served as control. The method used here well has limitations because wavy vessels may be cut several times. To exactly follow one and the same vessel in a given tumor, other methods are required. However, the aim of our study was to answer the question whether the assessment of tumor-associated blood vessel density with the means of a “routine” method applicable by dermatopathologists might be helpful to discriminate different types of BCCs from their histological simulators. 

 In contrast to normal skin, significant variations of blood vessel densities were found in the peritumoral stroma of different tumor entities, where the highest counts were present in BCCs and significantly lower counts in the peritumoral stroma of benign tumors such as trichofolliculomas, trichoepitheliomas, and trichoblastomas. The latter two may lead to differential diagnostic difficulties: in particular, desmoplastic trichoepithelioma may be confused with morphoeic BCCs and trichoblastoma with nodular BCCs. Our results show that determination of peritumoral microvessels might serve as an aid for differential diagnosis. VEGF, the main proangiogenetic cytokine, has been found increased in BCCs compared to normal skin, although to a lesser extent than in cutaneous squamous cell carcinoma (SCC) [20]. VEGF in BCCs was shown to be correlated to blood vessel density [21]. Our data showing a strikingly low intratumoral vessel density in all tumors investigated are in line with earlier reports comparing BCCs and SCC [10–12]. What means the paucity of small blood vessels within the epithelium of nodular BCCs compared to the plethora of those in the peritumoral stroma biologically? Some authors attribute intratumoral vessel formation to metastatic capacity of SCC in contrast to BCCs [12]. The peritumoral vessel density in BCCs was attributed to local aggressiveness [13, 22]. The focus of this study was the comparison between BCCs and their benign counterparts rather than comparison of variable vessel densities of more or less aggressive BCCs. Interestingly, the fibroepithelioma Pinkus displayed intermediate numbers of peritumoral vessels as compared to BCCs and compared to truly benign trichogenic neoplasms showing their “intermediate malignancy”. Are BCCs-associated blood vessels affected by the known pathogenic pathway of BCCs, the smoothened/Gli pathway activated due to absence/reduction of PTCH signalling? Translocations or mutations that are characteristic of distinct tumors have been detected in stromal cells as well [23]. A recent observation might disprove this in BCCs, since we observed in BCCs regressed during oral treatment with an inhibitor of the smo/gli pathway that in absence of tumor cells, the vessel-containing tumor bed was still present (data not shown). The question as to whether stromal fibroblasts in BCCs carry the PTCH mutation is, to our knowledge, not answered yet.

A number of studies have been performed to evaluate ancillary diagnostic tools that might differentiate BCCs from benign trichoblastic neoplasms. Immunohistochemical approaches to differentiate between these entities included the expression of androgen receptor, CD34, bcl-2, TGF-[beta], CD10, and staining for tumor-associated Merkel cells. Androgen receptor expression was detected in about 80% of basal cell carcinomas. Different from basal cell carcinomas, benign trichoblastic neoplasms showed no expression of androgen receptor [24]. CD34 was shown to be strongly expressed in the tumor-associated stroma of trichoepithelioma and absent or only focal in the dermis surrounding the nests of BCCs [25]. Bcl-2 expression was found diffuse in BCCs and mostly patchy and peripheral in trichoepithelioma [26]. TGF-[beta] staining is positive in trichoepithelioma and negative in most BCCs [27]. CD10 was postulated as useful for distinguishing between BCCs with widespread follicular differentiation and trichoblastomas [28]. Merkel cells are absent in most BBC and preferentially detectable in trichoepithelioma and trichoblastoma [29]. Cytokeratins have not been helpful, as their expression and staining pattern are generally similar in benign trichoblastic tumors and BCCs [29].

In conclusion, the data obtained in a large series of benign and malignant trichogenic tumors show that determination of peritumoral small vessel density by CD31 immunohistology adds reliable information to histological differential diagnosis.

## Figures and Tables

**Table 1:** Age and sex distribution.

Tumor	Mean age ± SD	Range	Male	Female
Nodular BCCs (*n* = 35)	75,1 ± 12,1	46–97	20 (57,1%)	15 (42,9%)
Morphoeic BCCs (*n* = 15)	76,1 ± 9,5	56–93	11 (73,3%)	4 (26,7%)
Pinkus' tumor (*n* = 17)	60,6 ± 13,6	32–80	5 (29,4%)	12 (70,6%)
Trichoblastoma (*n* = 20)	57,0 ± 18,0	21–91	15 (75,0%)	5 (25,0%)
Trichofolliculoma (*n* = 8)	56,4 ± 20,2	23–86	1 (12,5%)	7 (87,5%)
Trichoepithelioma (*n* = 11)	46,7 ± 17,7	25–79	3 (27,3%)	8 (72,7%)
Desmoplastic Trichoepithelioma (*n* = 6)	47,2 ± 17,4	18–64	1 (16,7%)	5 (83,3%)

**Table 2:** Location of tumors.

Location	Nod BCCs	Morph BCCs	P	TB	TF	TE	DTE
Capillitium	1			9	2	1	
Face							
Cheek	6	3			3	1	3
Forehead	4	3		2	1	1	1
Chin						1	
Temple	5	2					1
Nose	8	5		1	2	5	
Ear	1						
Retroauricular				1			
Chest and back	2	1	8	4		1	
Neck	4						
Shoulder	1		1	1		1	1
Abdomen			4				
Limbs	3	1	4	2			

Total	35	15	17	20	8	11	6

Nod BCCs: nodular basal cell carcinoma. Morph BCCs: morphoeic basal cell carcinoma. P: Pinkus' tumor. TB: trichoblastoma. TF: trichofolliculoma. TE: trichoepithelioma. DTE: desmoplastic trichoepithelioma.
